# Yoga as a therapeutic modality for psychological trauma in women and children in the Meitei-Kuki conflict in Manipur

**DOI:** 10.3389/fpsyg.2025.1682034

**Published:** 2025-10-16

**Authors:** R. K. Roshni Raj Lakshmi

**Affiliations:** Department of Yoga, Manipur University, Manipur, India

**Keywords:** children, conflict, mind-body medicine, psychological trauma, women, yoga

## Introduction

Conflict is “the disagreement or difference of opinions between or among individuals that can be potentially harmful to any organization” (Foncubierta-Rodríguez et al., 2023). This disagreement can be in an individual, between individuals, groups, an organization, nations, or political entities. Healthy resolution of conflict is necessary to achieve growth and harmony in any setting. Unresolved conflicts, especially between political bodies or countries, can escalate to warlike conditions, wreaking havoc in the affected regions. These conflicts harm the people of the region, especially the vulnerable group- the women and children.

## Meitei-Kuki conflict

Manipur, a north-eastern state of India, is ridden with conflict due to identity-based conflict of varied communities (Oinam, 2015). The evolving geopolitics of the region has further complicated the situation with entry of new entities like illegal immigrants from Myanmar, narco-terrorists and illegal outfits. The Meitei-Kuki conflict started in May 2023 following a rally taken out by All Tribal Students Union of Manipur (ATSUM) in the hill districts of Manipur (Bobichand, 2023). The 2-year-long ongoing conflict has adversely affected the law and order, economy, and fundamental human rights of its people. Inhuman acts have been conducted by both the warring groups, inflicting an indelible psychological trauma on the women and children, more specifically (Staff, 2023; Devi et al., 2025).

## Impact of the conflict on women and children

Conflicts and violence deleteriously affect the people of the affected regions. Often, the vulnerable and weak in the group are used as tools of oppression and subjugation by the warring groups. Throughout history, it has been seen that women and children are brutally tortured to show terror to the opponent (Jansen, 2006). Access to food, shelter, safety, medical facilities, and education is unavailable in most conflict-ridden places (Sharma, 2023). Armed conflict adversely affects child and maternal health causing drastic increase in mortality rates (Jawad et al., 2021).

## Psychological trauma of women and children

“Psychological trauma is a stressful event that causes distress that exceeds an individual's ability to integrate the emotions and cognitions involved in the experience” (van der Kolk et al., 1989). An individual feels helpless, disempowered, and disconnected from others when they are undergoing psychological trauma. There is a lack of social systems of care, protection, and meaning essential for life. During conflicts, people in the region experience traumatic events like the destruction of their homes, the brutal killing of people, sexual assault, and the forceful eviction from their homes. These events leave an ineradicable mark on the minds of the affected.

Women are often targeted by the opposing forces to create terror (terror (Comas-Díaz and Jansen, 1995). In armed conflicts, it is the women and children who bear the brunt of the struggle. Sexual assaults, killings, hostage holding are used as means to subjugate the opponent as was seen in Meitei-Kuki conflict also (NDTV, 2024). Children are not only collateral damage but are targeted by combatants to instill fear (Shenoda et al., 2018). Each and every system of the body is affected severely by the injuries leading to long-term repercussions. Children are strongly affected by their lived experiences, and exposure to violence can have a long-term impact on their personality and lifestyle (Guerra et al., 2003). Research has demonstrated that psychological trauma exposure during childhood can lead to problematic psychiatric conditions like dissociative personality, depression, aggression, and psychopathology in later life (Deputy et al., 2022; Slone and Shoshani, 2022).

## Possible remedies

The best solution to the problem is restoring peace to the region and regaining normalcy. Though the central government charts out political dialogues and conflict resolution, the resolution is still a far cry in reality. Meanwhile, the affected people need to be provided with coping strategies, emotional resources, and support to overcome the psychological trauma they have undergone, more so for the women and children. Efforts have been made to address the issue using mind-body medicine techniques like yoga as an add-on to provide some solace to the affected (Devi et al., 2025).

## Yoga as a therapeutic modality for affected women and children

Yoga, an ancient science, teaches an individual how to transcend ordinary existence and move to a higher level of consciousness (Bryant, 2009). Scriptures have defined yoga as a means to attain equanimity of mind (Tapasyananda, 2012). Today, it has evolved into a form of mind-body medicine that is highly effective in enhancing physical and mental health. It comprises practices like postures, breathwork, cleansing techniques, meditation, relaxation techniques, dietary regulations, and ethical principles, among many others. Studies have shown that yoga practices help in addressing the issues of people suffering from conditions like post-traumatic stress disorder (PTSD; Jindani et al., 2015; Cramer et al., 2018). Besides the conventional therapy for PTSD, yoga proves to be an effective add-on. It helps to regulate the autonomic nervous system, primarily by increasing parasympathetic dominance through practices like breathwork (Nolan, 2016). Slow and rhythmic breathing stimulates the vagal nerve and activate the PNS (Brown and Gerbarg, 2005). Studies have shown that yoga reduces cortisol, increases GABA and reduce stress reactivity (Nolan, 2016). Hence, the current article posits that yoga can be a valuable add-on to the conventional methods for psychological trauma faced by women and children in the Meitei-Kuki conflict in Manipur.

Yoga, a community activity, helps distract the individual from sitting alone and ruminating about the traumatic incidents. Practices like breathwork and relaxation induce a calm state of mind by increasing the vagal tone (Goldstein et al., 2016). Meditation helps downregulate the hyper-aroused hypothalamic-pituitary-adrenal and sympathetic-adrenomedullary axes through a top-down mechanism (Lemay et al., 2019). Physical stretches and asanas (postures) help to distract the mind from being aloof and introduce connectivity to others and an awareness of one's body. Simple practices like OM chanting reduce activation of the amygdala, which is responsible for negative emotions (Gangadhar et al., 2011). Studies demonstrate that yoga improves heart rate variability, reduces inflammatory markers, reduces fasting blood glucose and lipid profile indicating reduction in stress response of the body and influencing epigenetic change (Pascoe and Bauer, 2015). Yogic counseling is an important component in rewiring the individual's thinking process to a more positive and resilient outlook (Bhide et al., 2023). Yoga reduces the activation of the stress response for the same duration. Yoga is proven to reduce the allostatic load and introduce dominance of the parasympathetic nervous system (Khattab et al., 2007), thereby enhancing health. Taylor et al. emphasize that yoga and mindfulness-based interventions adjunctive to treatment as usual are effective in ameliorating symptoms of psychological trauma (Taylor et al., 2020). Hirsh and Ben-Arie state that the synergistic effect of mindfulness, yoga and support group improved the ability of conflict affected individuals to handle stress (Litvak Hirsch and Kassif Ben-Arie, 2025). Haller et al. showed that yoga with Cognitive Behavioral Therapy significantly reduced PTSD symptomatology (Haller et al., 2023).

Besides the short-term benefits of yoga on mental health and stress in particular, the long-term benefits can be demonstrated by studies on yoga and epigenetics (Kripalani et al., 2022). Rhodes et al. demonstrated that yoga has beneficial long-term effect on mental health of women with PTSD (Rhodes et al., 2016). Mitchell et al. showed in their study that yoga can be a useful tool for patients with PTSD who do not accept conventional therapy. Also, they emphasized that yoga was beneficial for women with PTSD (Mitchell et al., 2014). Nolan has stated that trauma sensitive yoga reduces PTSD symptomatology in women with PTSD as an adjunctive therapy (Nolan, 2016). Yoga is a subtle but powerful healing method that can have lasting effect on overall health of an individual.

Trauma can be of different types and can affect children and adults in multiple ways. Therefore, there is a need to design tailor-made yoga modules for a certain group of people, especially children. Gender is another factor that needs to be taken into consideration while designing the modules. As mentioned earlier, women and children are intentionally targeted to create terror. Certain practices involving touch may cause re-traumatization and hence, due care should be taken while preparing and implementing the module. Trauma sensitive/trauma informed yoga can be employed to ensure intervention for the special population without causing re-traumatization. TSY include “aspects of safety, invitational language, breathing techniques, choice, bodily autonomy, verbal cues versus physical assist, refraining from triggering postures, and promoting interoception” (Klukan and Lunsford, 2024). Most forms of yoga Vinyasa, Kundalini, and hatha yoga can be remodeled keeping the TSY cues in mind. Practices like breathwork, guided relaxation, yoga nidra, postures can be incorporated keeping the participants in mind.

## Challenges in implementation

Challenges and practical problems can be many while trying to give yoga interventions for women and children affected by psychological trauma during the armed conflict. Problems like restrictions on movement due to state imposed curfews, safety concerns, lack of weather-proof halls or classrooms for practice, lack of privacy during counseling, and difficulty in passing information due to the state-imposed internet ban. Yoga experts trained by reputed institutes are eligible for training for specific interventional study. Another challenge to intervene with yoga is due to probable cultural bias in the heterogenous cultural scenario in Manipur. Module preparation has to be done with an eye on this factor.

With all the problems withstanding, improvisations can be done to reach out to the affected people and provide the resources they need to cope with the situation. Deploying trained instructors who live close to the relief centers to implement the common protocol during curfew relaxation hours can be a means to provide yoga to the affected.

## Conclusion

In conclusion, yoga can be an effective and easily accessible method that can be used as an add-on to conventional approach to overcome psychological trauma in women and children in the Meitei-Kuki conflict in Manipur. Efforts should be made to ascertain the speculated benefits by conducting robust empirical studies with suitable designs. Policy needs to be made to make the services available to the affected people by integrating such services with the government aids or with collaboration with Non-Government Organizations.
